# Effects of Adding Antioxidants on the Lightfastness Improvement of Refined Oriental Lacquer

**DOI:** 10.3390/polym13071110

**Published:** 2021-03-31

**Authors:** Kun-Tsung Lu, Jia-Jhen Lee

**Affiliations:** Department of Forestry, National Chung Hsing University, 250 Kuo-Kuang Rd., Taichung 402, Taiwan; jessie135kimo@livemail.tw

**Keywords:** refined oriental lacquer, antioxidants, lightfastness, coating and film properties

## Abstract

Refined oriental lacquer (ROL) is a natural polymeric material with a satiny texture, elegant beauty, and high durability for wood furniture and handicraft finishing. However, its poor lightfastness, which results from the photo-degradation or photo-oxidation of its main component, catechol derivatives, must be improved for its widespread utilization. In this study, two experiments were performed. First, five types of antioxidants, including three primary antioxidants, such as 2,2′-methylenebis(6-nonyl-p-cresol) (coded as AO-1), 2,2′-methylenebis(6-tert-butyl-4-methylphenol) (AO-2), and bis [4-(2-phenyl-2-propyl) phenyl] amine (AO-N), and two secondary antioxidants, such as tris (2,4-ditert-butylphenyl) phosphite (AO-P) and dilauryl thiodipropionate (AO-S), were investigated to determine which is the most effective for improving the lightfastness of ROL. Secondly, the appropriate quantity of the best antioxidant, including 0, 1, 2, 3, 5, and 10 phr, was also determined. The lightfastness parameters, such as brightness difference (ΔL*), yellowness difference (ΔYI), and color difference (ΔE*), as well as other coating and film properties, were assessed. The results showed that the primary antioxidants had higher efficiency than secondary antioxidants for improving the lightfastness of ROL. Among the primary antioxidants, the 5 phr AO-N was the most effective at improving the lightfastness of ROL; however, 1 phr addition had already shown significantly improved efficiency. In addition, the drying time of ROL was extended and film properties decreased when increasing the content of AO-N, but the 1-phr-containing ROL displayed superior film properties, especially adhesion and bending resistance, compared with the raw ROL film.

## 1. Introduction

Oriental lacquer is a natural polymeric material with a water in oil (W/O) emulsion sap, which is obtained by tapping *Rhus* trees, specifically *Rhus succedanea*, found in Taiwan and Vietnam [1,2]. The sap of raw oriental lacquer must be refined by stirring under a temperature below 45 °C to reduce the water content to around 3% [3,4] or other refined processes [5,6,7,8] for shortening the curing time and improving the gloss and other film properties of the raw oriental lacquer. It is defined as a refined oriental lacquer (ROL). The ROL film possesses a wax-like gloss and has elegant beauty and high durability compared to synthetic coatings and is widely used for wood furniture and handicraft finishing in Taiwan. However, the poor lightfastness of ROL is a major limitation and it must be improved for the widespread utilization of ROL as a natural coating.

The poor lightfastness of ROL results from the main component, catechol derivatives, such as urushiol, laccol, or thitsiol, of *Rhus vernicifera* (grown in China and Japan)*, R. succedanea* (in Taiwan and Vietnam), and *Melanorrhoea usitata* (in Thailand and Myanmar) saps, respectively [2,3]. The catechol derivatives have a 15 or 17 carbon side chains at meta- or para-positions of the ortho-dioxy-benzol ring and with 0–3 unsaturated double bonds—namely, 3-pentadecatrienylcatechols for urushiol, 3-heptadecatrienylcatechols for laccol, and 4-heptadecatrienylcatechols for thitsiol. The catechol derivatives are dried first by the laccase-catalyzed dimerization of the ortho-dioxy-benzol and then aerobic auto-oxidative polymerization of the unsaturated side chains to form a network structure [1,3]. It is known that ultraviolet (UV) radiation provides strong energy to destroy the polymer materials by way of photo-degradation or photo-oxidation to produce excited state, free radical, peroxide, and singlet oxygen, etc. [9,10].

The catechol derivatives containing ROL coating networks were first photo-oxidated to form polymer oxy radicals (RO˙) and polymer peroxy radicals (ROO˙) under UV exposure and then the hydroxy (OH) and hydroperoxide (OOH) groups are formed by reacting these radicals with the same or neighboring polymer molecules (RH), respectively. Furthermore, the carbonyl groups, such as ketone groups, are formed by the internal rearrangement of polymer alkoxy ally biradicals in the side chain of catechol derivatives [11]. In addition, cracking, chalking, gloss loss, and white spots are found on the film’s surface after UV exposure. These phenomena are due to the photo-degradation and cause a plant gum, which is another component of oriental lacquer resulting from the coating network structure [2,12,13].

Many photo-stabilizers, such as UV screeners, UV absorbers, hindered amine light stabilizers (HALS), antioxidants, singlet oxygen scavengers, and excited state quenchers, can be used for reducing the photo-degradation of polymer films [9,14,15,16,17]. Hong et al. [11] added HALS and a benzotriazole UV absorber to an oriental lacquer to enhance its photo-stabilization. In our previous reports [12,13,18], effects of a titanium dioxide (TiO_2_) UV screener and different types of HALS addition on the lightfastness improvement of a refined oriental lacquer were examined. In this study, the antioxidant additions were further investigated for improving the lightfastness of ROL. Five types of antioxidant were used, including primary antioxidants such as hindered phenol, whose function is to scavenge peroxy radical intermediates (ROO˙) in the photo-oxidation process [19]. Another is secondary antioxidants such as phosphite, which is frequently referred to as a hydroperoxide (OOH) decomposer and acts to convert hydroperoxides into nonradical, nonreactive, and thermally stable products [20].

## 2. Materials and Methods

### 2.1. Materials

The oriental lacquer, harvested from the cultivar *R. succedanea*, was purchased from the Long-Nan Museum of Natural Lacquer Ware in Nantou, Taiwan. The composition of oriental lacquer was as follows: 54.1 ± 0.3% of laccol, 7.2 ± 0.4% of plant gum and laccase, 4.4 ± 0.7% of nitrogenous compounds, and 34.3 ± 0.1% of water. It was analyzed in our laboratory according to the CNS 2810 Standard [21].

**Figure 1:**
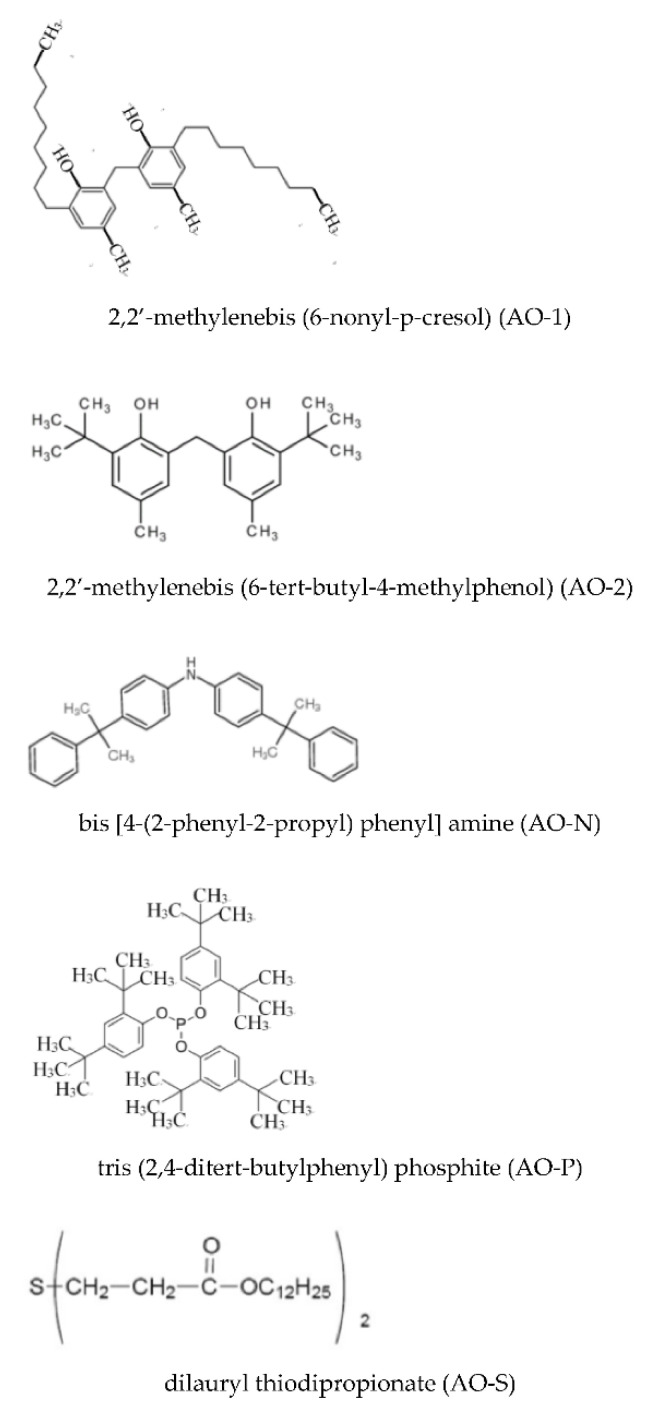
The structures of the five types of antioxidants.

Five types of antioxidant were used, including three primary antioxidants, such as 2,2′-methylenebis(6-nonyl-p-cresol) (coded as AO-1), 2,2′-methylenebis(6-tert-butyl-4-methylphenol) (coded as AO-2), and bis [4-(2-phenyl-2-propyl) phenyl] amine (coded as AO-N), and two secondary antioxidants, such as tris (2,4-ditert- butylphenyl) phosphite (coded as AO-P) and dilauryl thiodipropionate (coded as AO-S). All antioxidants were purchased from Easchem Co. Ltd., (Taipei, Taiwan), with the exception of the AO-N antioxidant, which was donated by Eutec Chemical Co. Ltd., Taiwan. The antioxidants were used as received without further purification. Figure 1 shows the structures of all antioxidants.

*Cryptomeria japonica* boards (with a moisture content of 11.0%, radial section), glass sheets, S-16 wear-resistant steel plates, tin-coated iron sheets, and Teflon sheets were used as substrates for specified experiments. All of the substrates were pretreated according to the CNS 9007 Standard [22].

### 2.2. Preparation of ROL

First, 400 g of oriental lacquer was placed in a glass container and stirred at 60 rpm, at 40 °C, with the mixing blades 5 mm from the bottom of the container, until the water content was reduced to 3.5% and the ROL was obtained.

### 2.3. Preparation of Antioxidant-Containing ROL

For determining the best antioxidant regarding the lightfastness of ROL, the different antioxidants, AO-1, AO-2, AO-N, AO-P, and AO-S, with 2 phr (by the solid content of ROL) were dissolved in 10 mL xylene and then added into the ROL, respectively. The mixtures were stirred evenly at 120 rpm for 10 min. The lightfastness of the antioxidant-containing ROL was evaluated and the best antioxidant selected. Finally, for determining the most appropriate quantity of antioxidant to add, amounts of 0, 1, 2, 3, 5, and 10 phr were added into the ROL, respectively. The preparation process was the same as that mentioned above and the coating and film properties were investigated.

### 2.4. Measurement of Coating Properties

The pH value was determined at 25 °C using a Suntex sp-701 probe (Suntex Instruments, Taipei, Taiwan). The viscosity was measured at 25 °C by a Brookfield DV-E Viscometer (Brookfield Engineering Laboratories, Middleboro, MA, USA). The drying time was measured at 25 °C, 80% RH, and with a wet film thickness of 76 µm using a three-speed BK Drying Time Recorder (BYK Additives & Instruments, Wesel, Germany). The drying states of touch-free dry (TF) and hardened dry (HD) were recorded as described by Lu et al. [23] and Chang et al. [4].

### 2.5. Preparation and Determination of Film Properties

The selected substrates were finished with different ROLs using a universal applicator (B-3530, Elcometer, Manchester, UK) with a wet film thickness of 100 µm. The specimens were first placed in a chamber with a constant temperature of 25 °C and humidity of 80% RH for one day, followed by placing them in an air-conditioned environment of 26 °C and 65% RH for another week. The film properties were evaluated after this drying process.

The lightfastness of the film was evaluated using a Paint Coating Fade Meter (Suga Test Instruments Co., Tokyo, Japan) at a chamber temperature of 32 ± 4 °C and using a H400-F mercury lamp (SUGA Test Instruments Co., Ltd., Tokyo, Japan) as a light source. According to CIE L*, a*, b* color system, after 0, 12, 24, 48, 96, 144, and 192 h exposure, the color changes, including brightness difference (ΔL*), yellowness difference (ΔYI), and color difference (ΔE*), were obtained using a spectrophotometer (CM-3600d, Minolta. Osaka, Japan) measurement with a D_65_ light source, test angle of 10°, and test window diameter of 8 mm and computed from the Minolta MCS software system. Nine points were measured and averaged for each specimen. The scanning electron microscope (SEM) inspection was performed using an electron microscope (SM-200, Topcon, Tokyo, Japan) with a magnification of 1350×. The Fourier transform infrared spectroscopy (FTIR) analysis was conducted using a Perkin-Elmer Spectrum 100 instrument (Perkin-Elmer, Shelton, CT, USA) with single-spot attenuated total reflection (ATR) at a range of 4000 to 650 cm^−1^ at a resolution of 4 cm^−1^ and averaged over 16 scans for each specimen.

The hardness of the film on glass sheets was evaluated according to the DIN 53157 using a König/Persoz Pendulum Hardness Tester (Braive Instruments, Liège, Belgium). Seven points were measured and averaged for each specimen. The mass retention was determined by placing approximately 0.3 g free film, which was separated from the Teflon sheet into a Soxhelt extractor (Dogger Co., New Taipei City, Taiwan) containing 250 mL acetone. The solution was siphoned four times per hour (total 6 h), and the soaked film was dried in an oven at 50 °C for 6 h and the weight retention was calculated. Five repetitions were performed and averaged. For determining the glass transition temperature (Tg) of film, dynamic mechanical analysis was carried out with a Perkin-Elmer DMA 8000 (Perkin-Elmer, Waltham, MA, USA) using tension mode at a frequency of 1 Hz, temperature of 0–180 °C, and a heating rate of 5 °C min^−1^ in a nitrogen atmosphere.

The impact resistance of the film on wood was investigated based on the falling weight of 300 g and an impact needle diameter of 1/2 inch by using the DuPont Impact Tester IM-601 (DuPont Co., Wilmington, DE, USA) and the height of the intact film was recorded. The adhesion of film on wood was evaluated by using the crosscut method according to CNS 10756 K 6800 [24]. The best adhesion is Grade 10, followed by Grades 8, 6, 4, 2, and 0. The bending resistance of film on tin-coated iron sheet was carried out according to JIS-K-5400 [25] by using a bending tester (Ueshima Seisakusho Co., Ltd., Tokyo, Japan) with steel bars with diameters of 2, 3, 4, 6, 8, and 10 mm. The tensile strength and elongation at break were evaluated according to ASTM D-638 Standard [26] by using a Shimadzu EZ Test Series Tensile Tester (Shimadzu, Kyoto, Japan) at a speed of 5 mm min^−1^ and with a fixture distance of 40 mm. Seven samples were tested and averaged for each specimen. Thermogravimetric analysis (TGA) of film was conducted by using a Perkin-Elmer STA 6000 (Perkin-Elmer, Waltham, MA, USA) in a nitrogen atmosphere with the temperature increasing from 50 to 700 °C at a heating rate of 10 °C min^−1^.

## 3. Results and Discussion

### 3.1. Lightfastness of ROL Films with Different Antioxidants

The time-dependent brightness difference (ΔL*), yellowness difference (ΔYI), and color difference (ΔE*) of ROL films with different antioxidants after UV exposure test are listed in Table 1, Table 2 and Table 3, respectively. All of the ΔL*, ΔYI, and ΔE* values increased with increasing UV exposure time. However, the films containing primary antioxidants AO-2 and AO-N had ΔL* values of 2.7 and 0.5, ΔYI values of 45.7 and 22.3, and ΔE* values of 11.1 and 5.3 after a UV exposure time of 192 h, which are significantly lower than those of blank (ΔL*, ΔYI, and ΔE* of 12.3, 99.9, and 32.4) and other antioxidants. The results also showed that the primary antioxidants (AO-1, AO-2, and AO-N) had higher efficiency than secondary antioxidants (AO-P and AO-S) for improving the lightfastness of ROL. In principle, the photo-oxidation of catechol derivatives containing ROL could be stopped to form polymer oxy radicals (RO˙) and polymer peroxy radicals (ROO˙) under UV exposure if the primary antioxidants acted as efficient radical scavengers. Furthermore, the primary antioxidants also interrupted peroxy radicals (ROO˙) to abstract a proton from the same or neighboring polymer (RH) to form the hydroxy (OH) and hydroperoxide (OOH) groups [19,27].

In this study, bis [4-(2-phenyl-2-propyl) phenyl] amine (AO-N) was the most efficient for improving the lightfastness of ROL, followed by 2,2′-methylenebis (6- tert-butyl-4-methylphenol) (AO-2) and 2,2′-methylenebis(6-nonyl-p-cresol) (AO-1). This result is due to the -NH group of the amine antioxidant (AO-N) reacting more easily with peroxy radicals (ROO˙) than the -OH group of phenolic antioxidants (AO-1 and AO-2) and providing a more easily abstracted proton than those of the polymer (RH) to form a stable, sterically hindered structure, thus interrupting the further photo-oxidation of the ROL [19,27]. In addition, as the AO-1 possesses long alkyl chains, which reduce the reaction of -OH groups with peroxy radicals (ROO˙), it was inferior to AO-2 in improving the lightfastness of ROL.

Among the secondary antioxidants, dilauryl thiodipropionate (AO-S) had also an ability to improve the lightfastness of ROL. However, after long-term UV exposure, it lost the improving ability; for example, after more than 144 h UV exposure, the ΔL*, ΔYI, and ΔE* values of ROL were higher than those of the blank group. It could be elucidated that the secondary antioxidants acted as hydroperoxide (OOH) decomposers. The hydroperoxide was produced from the initial stage products, peroxy radicals (ROO˙), during photo-degradation; therefore, the improvement in the efficiency of the secondary antioxidant is inferior to that of the primary antioxidant. In addition, among the five types of antioxidants used in this study, the secondary antioxidant AO-P, which contained the phosphite group, was the least efficient for the lightfastness improvement of ROL. Thsi may be because the AO-P possesses three units of benzene structure, and the steric hindrance decreases the reactivity with ROOH, and a quinone structure is formed after UV irradiation. The result shows that the ROL with AO-P had the highest ΔL*, ΔYI, and ΔE* values.

### 3.2. Coating and Film Properties of ROL with Different Contents of Antioxidant

In Section 3.1, it is reported that the primary antioxidant of the bis [4-(2-phenyl-2-propyl) phenyl] amine (AO-N)-containing ROL film had the best lightfastness. Therefore, for determining the best content of AO-N, the coating and film properties of ROL were further investigated.

#### 3.2.1. Coating Properties of ROL

The coating properties of ROL with different contents of AO-N are listed in Table 4. The ROL with and without AO-N had the same pH values of 3.4–3.5, meaning that the activity of laccase was be affected by the addition of the antioxidant, and the optimum pH value of ROL with the best activity of laccase is 3.0~5.0 [11,12]. The viscosity of ROL increased with increasing amount of AO-N added. This result may be due to the –N-H group in AO-N, and the greater hydrogen bonding increases the viscosity of ROL. However, the viscosity of ROL from 0 phr of 113 cps increased to 10 phr of 148 cps. The slight increase in viscosity did not affect the finishing operation of ROL.

The drying time of ROL without AO-N was the touch-free dry (TF) of 5.0 h and hardened dry (HD) of 11.0 h whereas it extended with increasing content of AO-N. The ROL with 10 phr AO-N had the longest drying time of 6.0 and 20 h for TF and HD, respectively. It is known that the catechol derivatives of ROL are dried first by the laccase-catalyzed dimerization of the ortho-dioxy-benzol and then aerobic auto-oxidative polymerization of the unsaturated side chains to form a network structure. In the auto-oxidative polymerization stage, the formed hydro-peroxide radicals (ROO˙), which polymerize to form a network structure, are captured by the Ar-NH groups of AO-N to form a hydro-peroxide (ROOH) and undergo retarded auto-oxidative polymerization of ROL, exhibiting a longer drying time after adding AO-N.

#### 3.2.2. Lightfastness of ROL Films

**Figure 2:**
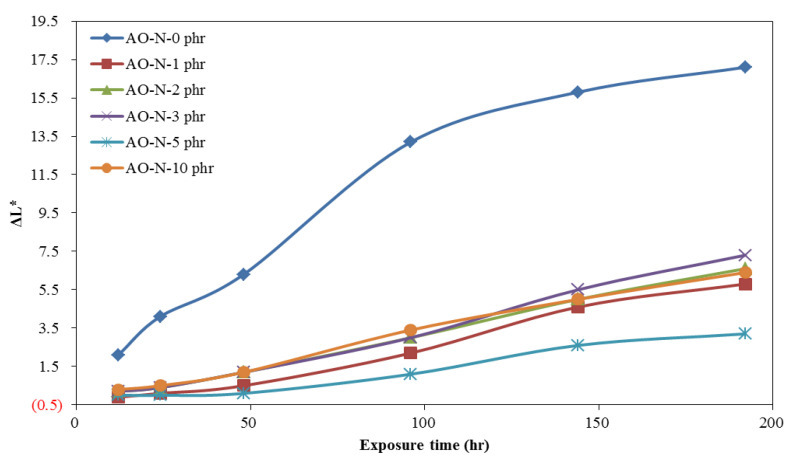
Time-dependent brightness difference (ΔL*) of ROL films with different contents of AO-N after UV exposure test.

**Figure 3:**
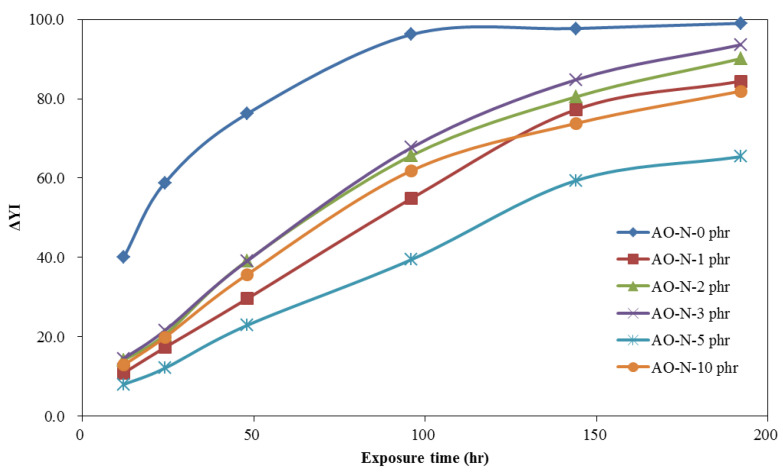
Time-dependent yellowness difference (ΔYI) of ROL films with different contents of AO-N after UV exposure test.

**Figure 4:**
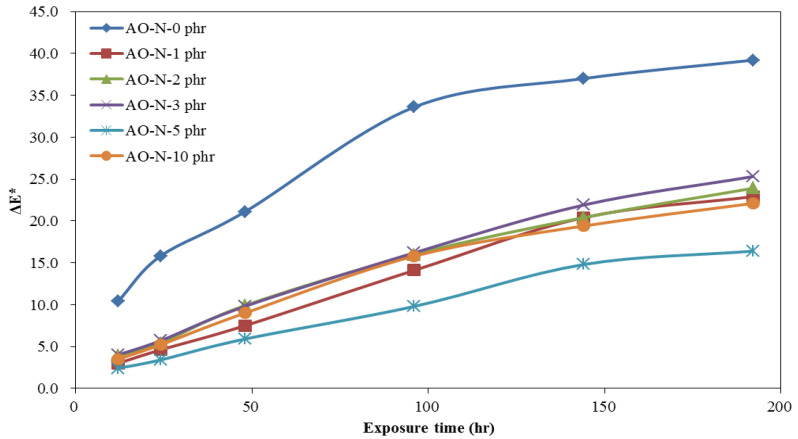
Time-dependent color difference (ΔE*) of ROL films with different contents of AO-N after UV exposure test.

The time-dependent brightness difference (ΔL*), yellowness difference (ΔYI), and color difference (ΔE*) of ROL films with different contents of AO-N after UV irradiation are shown in Figure 2, Figure 3 and Figure 4, respectively. The ΔL* of ROL films increased with prolonged exposure time under UV irradiation. This phenomenon results from the photo-degradation and photo-oxidation to destroy the network structure of ROL and white particle plant gum migrated to the surface [28]. The ROL film without AO-N (0 phr) had the largest change in the brightness of the film. The result showed that with only 1 phr addition, the ΔL* value could be lowered significantly; however, 5 phr content yielded the best improvement in the brightness of the ROL film. The yellowness difference (ΔYI) of ROL films also increased with increasing UV exposure time; this may be due to the chormophoric group of quinone structures formed from the benzene rings in the ROL film after photo-oxidation. The ROL film without AO-N yellowed seriously after only UV irradiation of 12 h, and a twofold increase in the ΔYI value after UV exposure of 48 h was found. The color difference (ΔE*) is a synergistic effect of ΔL* and ΔYI, and the trend was the same as the ΔL* and ΔYI.

The color change parameters of ΔL*, ΔYI, and ΔE* values after the lightfastness test of 192 h showed that the values of the ROL film without AO-N were 17.1, 99.0, and 39.2, respectively, and all of the values collected with different contents of AO-N were lower than those without AO-N. However, the decrease in the color change parameters was not proportionate to the content of AO-N, i.e., the ROL film with 5 phr AO-N had the lowest ΔL*, ΔYI, and ΔE* values of 3.2, 65.4, and 14.4, and with 10 phr, AO-N increased to 6.4, 81.9, and 22.1, respectively. In practice, the ΔL*, ΔYI, and ΔE* values of the ROL film with 1 phr AO-N were 5.8, 84.4, and 22.9, respectively, and it already demonstrated significant efficiency in improving the lightfastness.

#### 3.2.3. SEM Inspection of ROL Films

**Figure 5:**
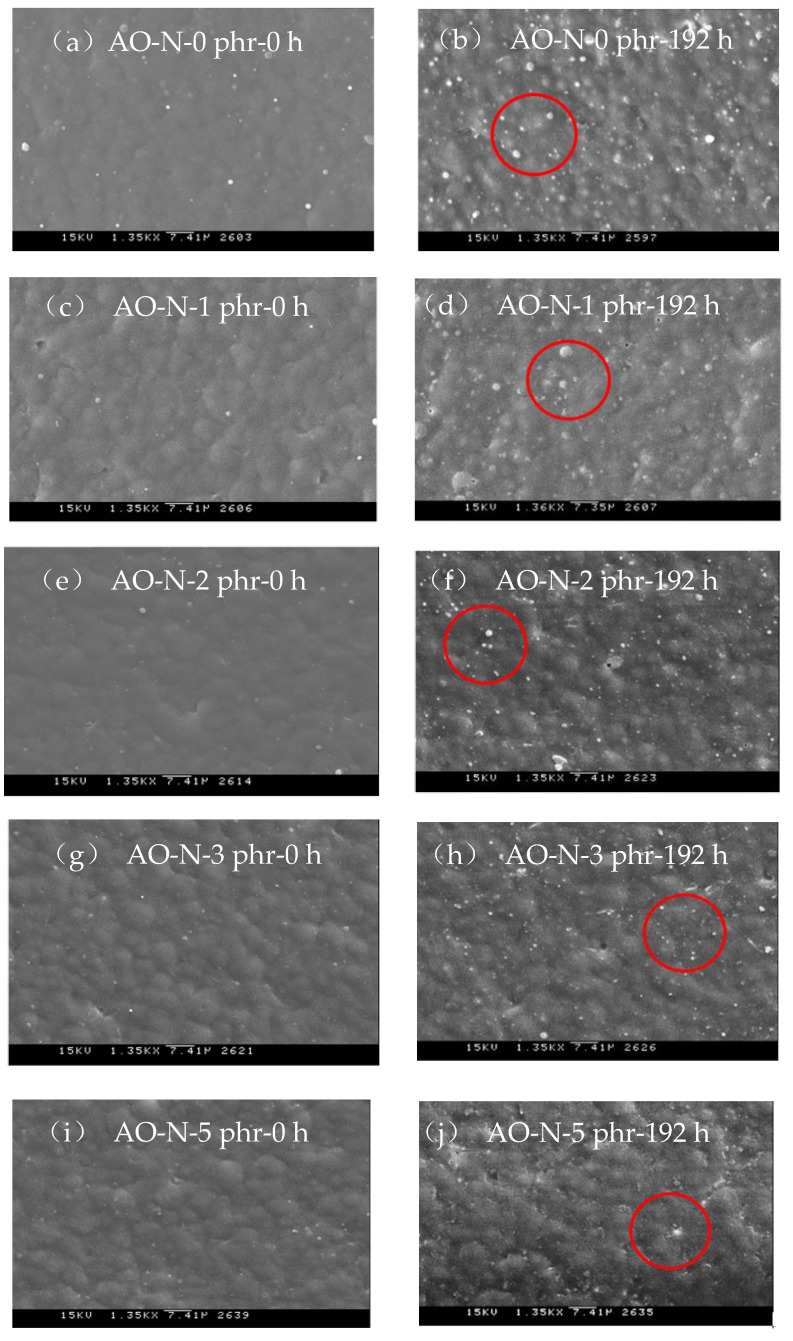

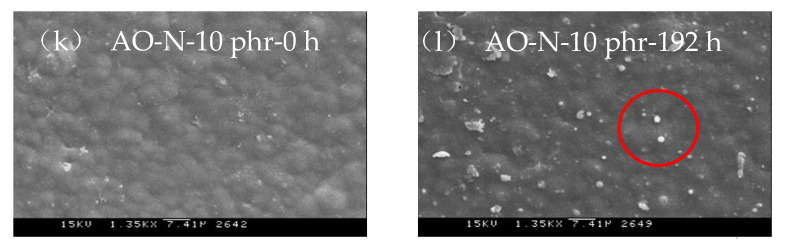
Scanning electron microscopic images (1350×) of ROL films with different contents of AO-N.

The SEM images of ROL films with different contents of AO-N before and after UV exposure of 192 h are shown in Figure 5. The ROL film without AO-N had more white particles of plant gum (as Figure 5b), which was due to severe photo-degradation under UV exposure [2,12,13,28]. However, the white spots were less prevalent in the ROL films with AO-N after UV irradiation. These results were also confirmed by the decreasing ΔL* values of the ROL films after adding the AO-N antioxidant, as shown in Figure 2.

#### 3.2.4. FTIR Analysis of ROL Films

**Figure 6:**
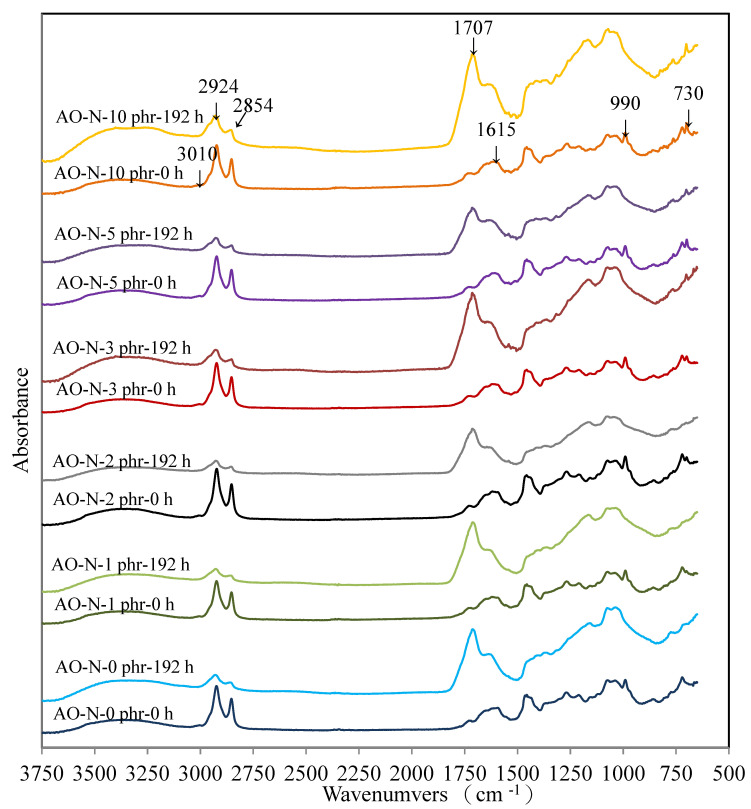
FTIR spectra of ROL films with different contents of AO-N.

The FTIR spectra of ROL films with different contents of AO-N before and after UV exposure of 192 h are shown in Figure 6. The functional groups of the ROL film without AO-N (0 phr) showed the -OH at 3200–3400 cm^−1^; -C-H stretching vibration of -C=C-H at 3010 cm^−1^; -CH_2_ asymmetric and symmetric stretching vibration of the urushiol side chain at 2924 cm^−1^ and 2854 cm^−1^, respectively; -C-O-C- stretching vibration at 1715 cm^−1^ and -C=O stretching vibration at 1700 cm^−1^; -C=C- stretching vibration and -C-H out of plane bending vibration of the urushiol benzene ring at 1615 cm^−1^ and 730 cm^−1^, respectively; the conjugated triene of the urushiol side chain also can be seen at 990 cm^−1^ [5,12,29]. The FTIR spectra of the ROL films with different contents of AO-N were similar to that of the ROL film without AO-N before UV exposure. However, after the 192-h lightfastness experiment, the peak at 3010 cm^−1^ disappeared and those at 2924 cm^−1^, 2854 cm^−1^, and 990 cm^−1^ decreased. The characteristic peaks at 1715 cm^−1^ (-C-O-C-) and 1700 cm^−1^ (-C=O) increased and consolidated in a new broad peak at 1707 cm^−1^. The results showed that the -CH bonding in the side chain fractured by photo-degradation and the peroxides formed. Furthermore, the degradation caused damage to the films and the plant gum migrated to the surface of the film [19,30]. In addition, the peak at 730 cm^−1^ also decreased due to the benzene ring degradation, and quinone compounds formed. From the FTIR analysis, it could be concluded that the photo-degradation of the ROL film was a result of the side chain and catechol structure of the network film. This result is also confirmed by the previous study by Hong et al. [11]. Figure 6 also shows that the ROL films with different contents of AO-N had a similar FTIR spectrum after UV exposure of 192 h, meaning that the peak at 3010 cm^−1^ disappeared, those at 2924 cm^−1^, 2854 cm^−1^, and 990 cm^−1^ decreased, and that at 1707 cm^−1^ increased. However, among the spectra, the 5-phr-AO-N-containing film showed the lowest increase in the peak at 1707 cm^−1^, demonstrating that it possessed the best lightfastness, and this result is also confirmed in Section 3.2.2.

#### 3.2.5. Film Properties of ROL

The fundamental film properties of ROL are listed in Table 5. The ROL film without AO-N had the highest hardness of 78 sec and the one containing 10 phr AO-N showed the lowest hardness of 67 s. The other films with AO-N content had a hardness of 72~75 s, which was slightly lower than that of the raw ROL film. The mass retention and Tg had the similar trends regarding the hardness. The raw ROL film had the highest mass retention and Tg of 93.7% and 103 °C, which decreased with increasing content of AO-N. In addition, the raw ROL film also had the best impact resistance of 10 cm, compared to only 5 cm for the other AO-N-containing films. The raw ROL and 1-phr-AO-N-containing films showed the best adhesion of Grade 10; however, there were no differences in bending resistance for all samples except the 10-phr-AO-N-containing film, which yielded a rather poor value of 10 mm. The results mentioned above show that the auto-oxidative polymerization of the unsaturated side chains of ROL was inhibited by adding the AO-N antioxidant, which decreased the cross-linking density of the network structure. The same trends were also found in tensile strength. The raw ROL film had a tensile strength and elongation at break of 16.5 Mpa and 10.8%, while the tensile strength decreased and the elongation at break increased with the addition of AO-N. The 10-phr-AO-N-containing film had the lowest tensile strength and elongation at break of 4.9 Mpa and 3.3% due to its low cross-linking density.

**Figure 7:**
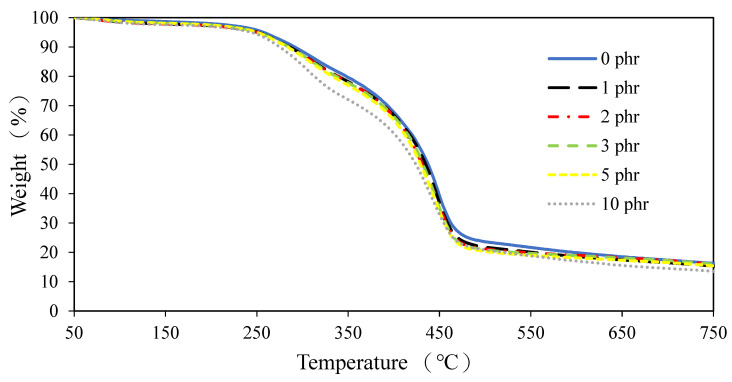
Thermogravimetric diagrams of ROL films with different contents of AO-N.

**Figure 8:**
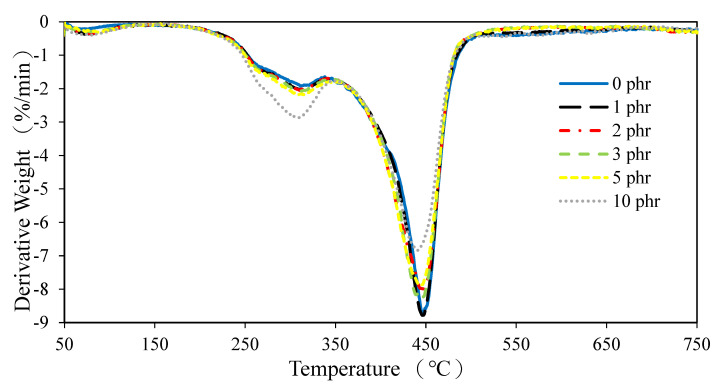
Derivative thermogravimetric (DTG) diagrams of ROL films with different contents of AO-N.

The TGA curves of ROL films with different contents of AO-N are displayed in Figure 7 and Figure 8, respectively. The ROL films with or without AO-N showed similar behavior in terms of thermal degradation. They consisted of three dominant steps at around 100–200 °C, 200–350 °C, and 350–550 °C, corresponding to the water vaporization and decomposition of the low-molecular-weight compounds, the degradation of the side chain of urushiol, and the decomposition of the network structure of the ROL film, respectively. This result is also confirmed by the previous study by Niimura et al. [1,6]. The results showed that in the second step, the raw ROL film had a temperature of maximum decomposition rate (T_max_) and derivative weight at T_max_ around 313 °C and −1.9 %.min^−1^. The other films with AO-N showed similar results, except for the 10-phr-AO-N-containing one, which had the largest derivative weight at T_max_ of −2.9%.min^−1^. In the third step, the T_max_ of raw ROL and 1-phr-AO-N-containing films was 446 °C, while those of the other films were slightly lower than the temperature; for example, that of the 10-phr-AO-N-containing film was 440 °C. The results showed that the thermal stability of the ROL film became inferior after adding AO-N and decreased with increasing content of AO-N due to the decrease in the cross-linking density of the network structure.

## 4. Conclusions

For improving the lightfastness of ROL, five types of antioxidants, including three primary antioxidants (AO-1, AO-2, and AO-N) and two secondary antioxidants (AO-P and AO-S), were used. The best antioxidant and most suitable amount of antioxidant were investigated. The results showed that the efficiency of lightfastness improvement of the primary antioxidants was higher than that of the secondary antioxidants. Among the primary antioxidants, bis [4-(2-phenyl-2-propyl) phenyl] amine (AO-N) was the best for improving the lightfastness of ROL. Among the different amounts of AO-N, the 5 phr addition was the best choices; however, 1 phr AO-N addition already showed a significant improvement efficiency. In addition, the drying time of ROL was extended and the film properties decreased with increasing amount of AO-N added. However, among the different AO-N-containing ROL films, the one with 1 phr showed superior film properties, especially regarding the adhesion and bending resistance, compared with the raw ROL film.

## Figures and Tables

**Table 1:** Time-dependent brightness difference (ΔL*) of refined oriental lacquer (ROL) films with different antioxidants after UV exposure.

Antioxidants (2 phr)	ΔL* after UV Exposure (h)					
	12	24	48	96	144	192
Blank ^a^	1.5	2.7	5.2	9.5	11.1	12.3
AO-1 ^b^	1.6	2.6	5.1	9.1	11.6	11.9
AO-2 ^c^	0.3	0.1	0.9	1.8	2.0	2.7
AO-N ^d^	−0.2	−0.4	−0.2	0.0	0.1	0.5
AO-P ^e^	2.2	4.4	7.7	12.7	15.1	16.6
AO-S ^f^	0.9	1.9	4.6	8.7	9.4	12.5

^a^ ROL without antioxidant; ^b^ 2,2′-methylenebis (6-nonyl-p-cresol); ^c^ 2,2′-methylenebis (6-tert-butyl-4-methylphenol); ^d^ Bis [4-(2-phenyl-2-propyl)phenyl] amine; ^e^ Tris(2,4-ditert-butylphenyl) phosphite; ^f^ Dilauryl thiodipropionate.

**Table 2:** Time-dependent yellowness difference (ΔYI) of ROL films with different antioxidants after UV exposure.

Antioxidants (2 phr)	ΔYI after UV Exposure (h)					
	12	24	48	96	144	192
Blank ^a^	32.4	49.2	72.4	91.3	98.2	99.9
AO-1 ^b^	34.3	49.8	69.7	92.3	98.3	101.4
AO-2 ^c^	8.3	11.5	19.8	31.9	38.7	45.7
AO-N ^d^	1.5	2.9	5.9	11.7	16.5	22.3
AO-P ^e^	39.6	63.2	82.4	97.9	102.0	102.8
AO-S ^f^	20.4	39.7	65.8	91.4	94.8	102.1

^a, b, c, d, e, f^ the same as Table 1.

**Table 3:** Time-dependent color difference (ΔE*) of ROL films with different antioxidants after UV exposure.

Antioxidants (2 phr)	ΔE* after UV Exposure (h)					
	12	24	48	96	144	192
Blank^a^	8.3	12.5	19.4	27.4	30.8	32.4
AO-1 ^b^	8.7	12.5	18.4	26.7	30.7	31.8
AO-2 ^c^	2.3	2.9	5.0	7.9	9.4	11.1
AO-N ^d^	0.6	0.9	1.5	2.8	3.9	5.3
AO-P ^e^	10.2	16.7	23.7	32.6	36.8	39.0
AO-S ^f^	5.3	10.1	17.4	26.6	28.0	33.0

^a, b, c, d, e, f^ the same as Table 1.

**Table 4:** Coating properties of ROL with different contents of AO-N.

Contents of AO-N (phr)	pH	Viscosity (cps, 25 °C)	Drying Time (h (25 °C, 80% RH)	
			TF ^a^	HD ^b^
0	3.5	113	5.0	11.0
1	3.5	116	5.0	12.5
2	3.5	129	5.5	13.5
3	3.4	131	5.5	13.5
5	3.4	136	6.0	15.0
10	3.5	148	6.0	20.0

^a^ TF: Touch-free dry; ^b^ HD: Hardened dry.

**Table 5:** Film properties of ROL with different contents of AO-N.

Contents of AO-N (phr)	Hardness (König, s)	Mass Retention (wt. %)	Tg (°C)	Impact Resistance (300 g, cm)	Adhesion (Grade)	Bending Resistance (mm)	Tensile Strength (MPa)	Elongation at Break (%)
0	78 ± 2	93.7 ± 0.8	103	10	10	6	16.5 ± 0.9	10.8 ± 1.0
1	72 ± 4	92.7 ± 1.4	98	5	10	6	15.4 ± 0.2	11.6 ± 1.3
2	74 ± 1	93.0 ± 1.4	97	5	8	6	15.3 ± 0.1	11.8 ± 0.9
3	74 ± 1	91.8 ± 0.3	95	5	6	6	13.9 ± 0.9	13.2 ± 0.0
5	75 ± 1	90.3 ± 0.6	88	5	6	6	12.3 ± 0.5	10.9 ± 0.7
10	67 ± 3	86.7 ± 0.8	86	5	6	8	4.9 ± 0.2	3.3 ± 0.3
